# Supplementary material for: Huisgen [3 + 2] Dipolar Cycloadditions of Phthalazinium Ylides to Activated Symmetric and Non-Symmetric Alkynes
Source: Molecules. 2020 Sep 25;25(19):4416. doi: 10.3390/molecules25194416 (PMC7582813; doi:10.3390/molecules25194416)
Supplement: Supplementary file 1 [file molecules-25-04416-s001.pdf]

Supporting information for:

Huisgen [3 + 2] Dipolar Cycloadditions of Phthalazinium Ylides to Activated Symmetric and Non-Symmetric Alkynes

Vasilichia Antoci [1], Costel Moldoveanu [1], Ramona Danac [1], Violeta Mangalagiu [2,*] and Gheorghita Zbancioc [1,*]

[1] Alexandru Ioan Cuza University of Iasi, Faculty of Chemistry, 11 Carol 1st Bvd, Iasi -700506, Romania; vasilichia.antoci@uaic.ro (V.A.); costel.moldoveanu@uaic.ro (C.M.); rdanac@uaic.ro (R.D.); gheorghita.zbancioc@uaic.ro (G.Z.)

[2] Alexandru Ioan Cuza University of Iasi, Institute of Interdisciplinary Research- CERNESIM Centre, 11 Carol I, Iasi, 700506, Romania; violeta.mangalagiu@uaic.ro (V.M.)

* Correspondence: gheorghita.zbancioc@uaic.ro (G.Z.); violeta.mangalagiu@uaic.ro (V.M.).

Contents

1. NMR Spectra of the obtained compounds. 2

1. NMR Spectra of the obtained compounds.

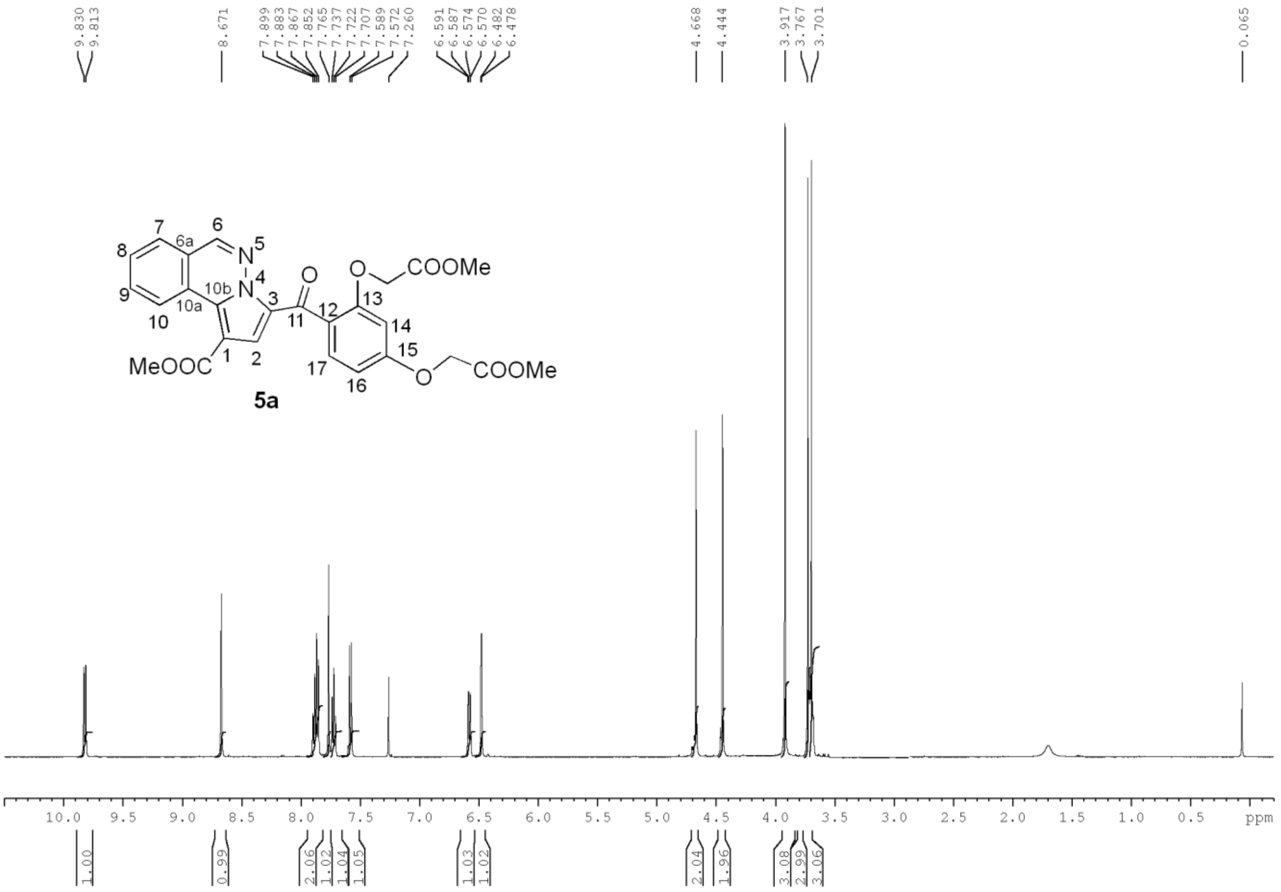

Figure S1: ^{1}H-NMR spectrum of the compound **5a.**

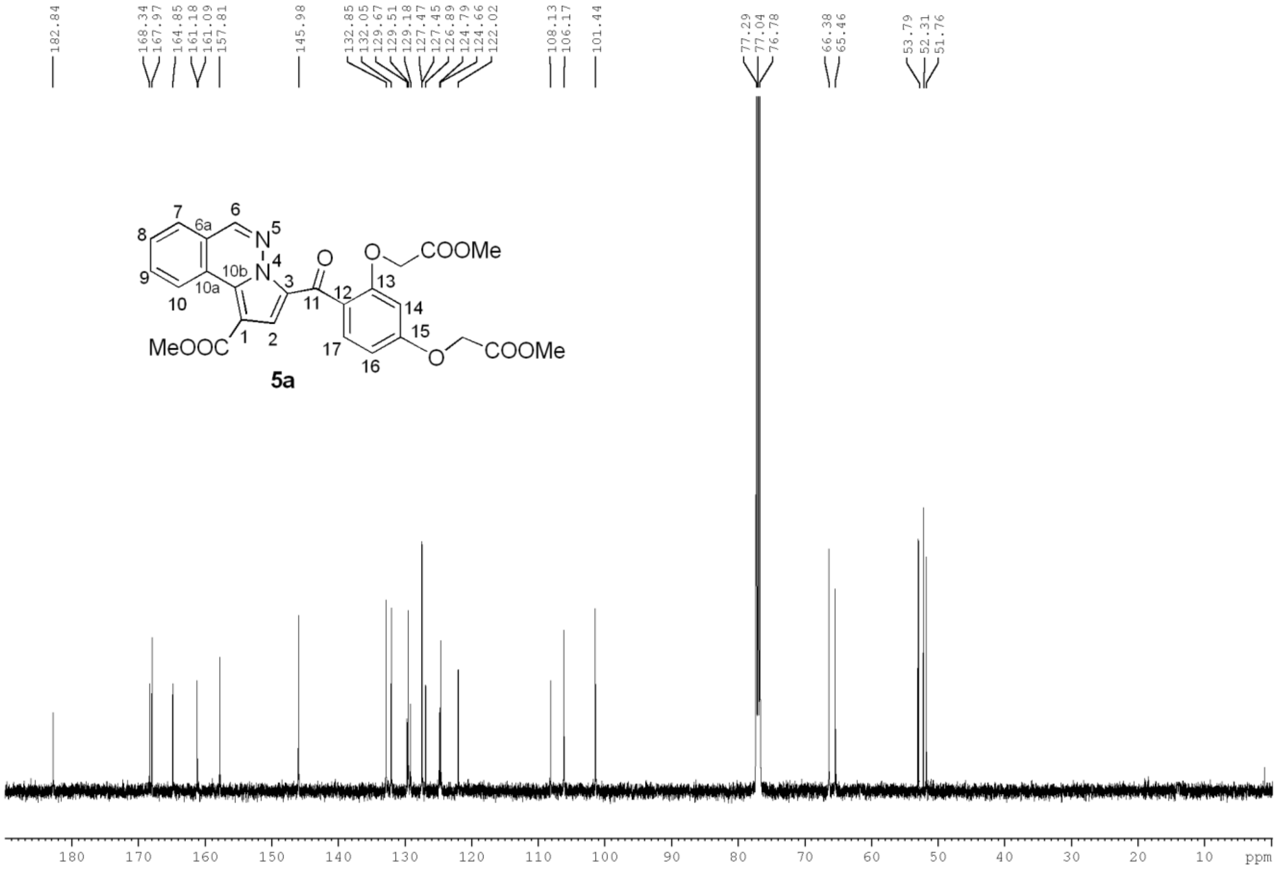

Figure S2: ^{13}C-NMR spectrum of the compound **5a.**

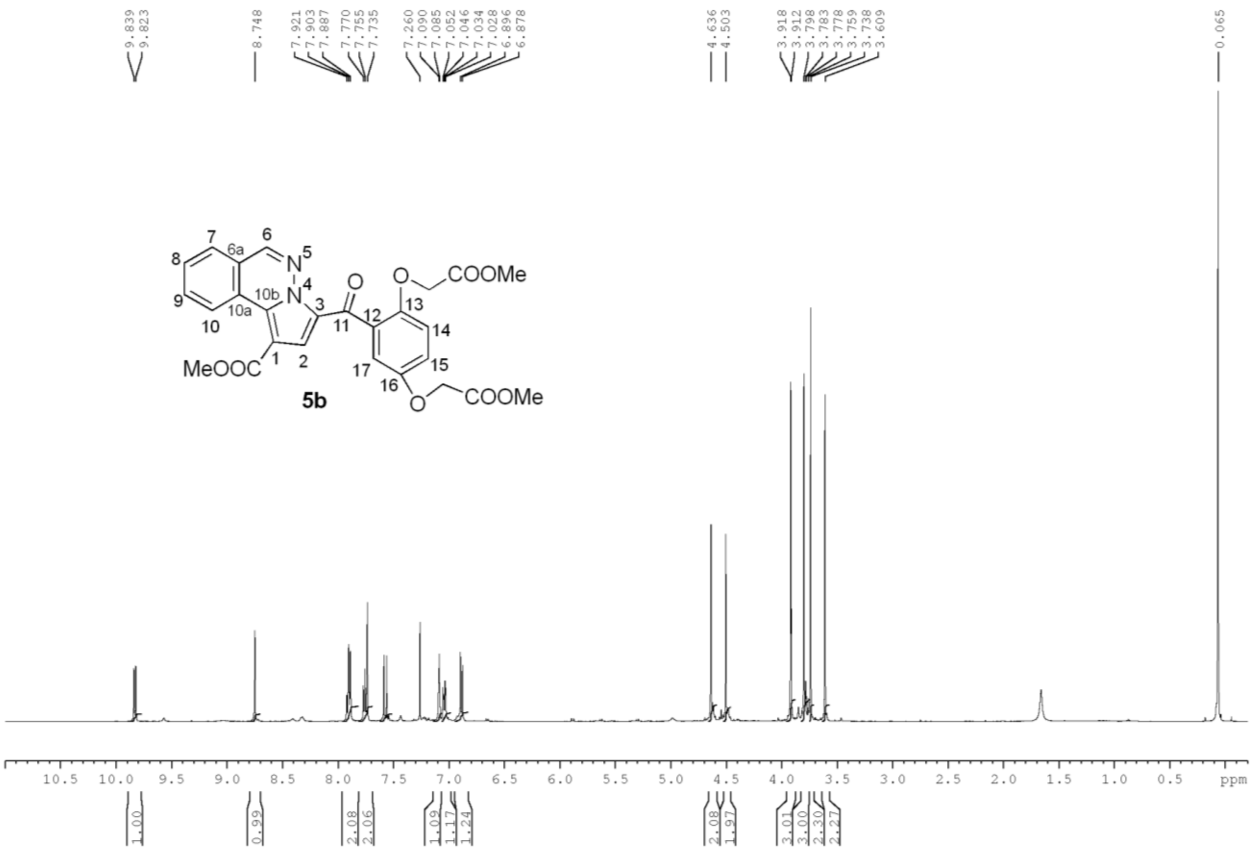

Figure S3: ^{1}H-NMR spectrum of the compound **5b.**

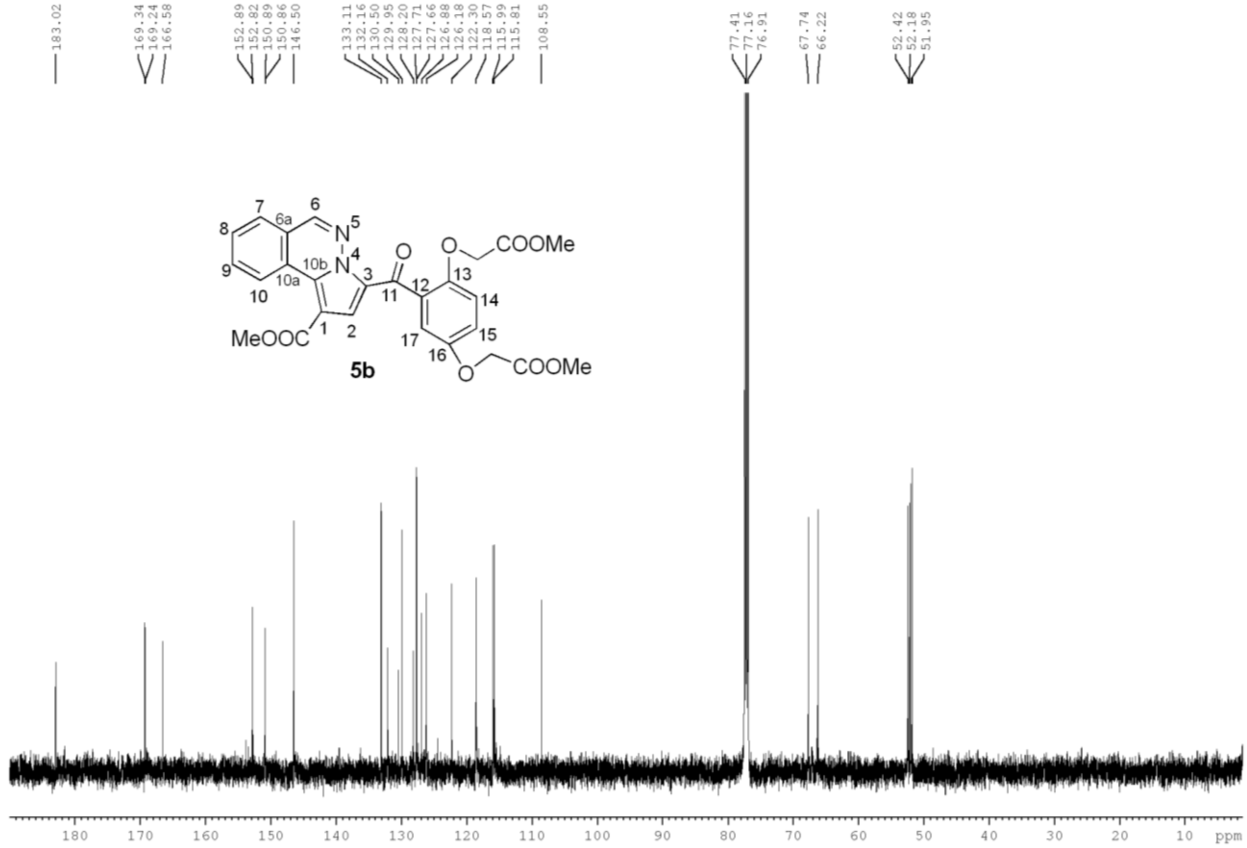

Figure S4: ^{13}C-NMR spectrum of the compound **5b.**

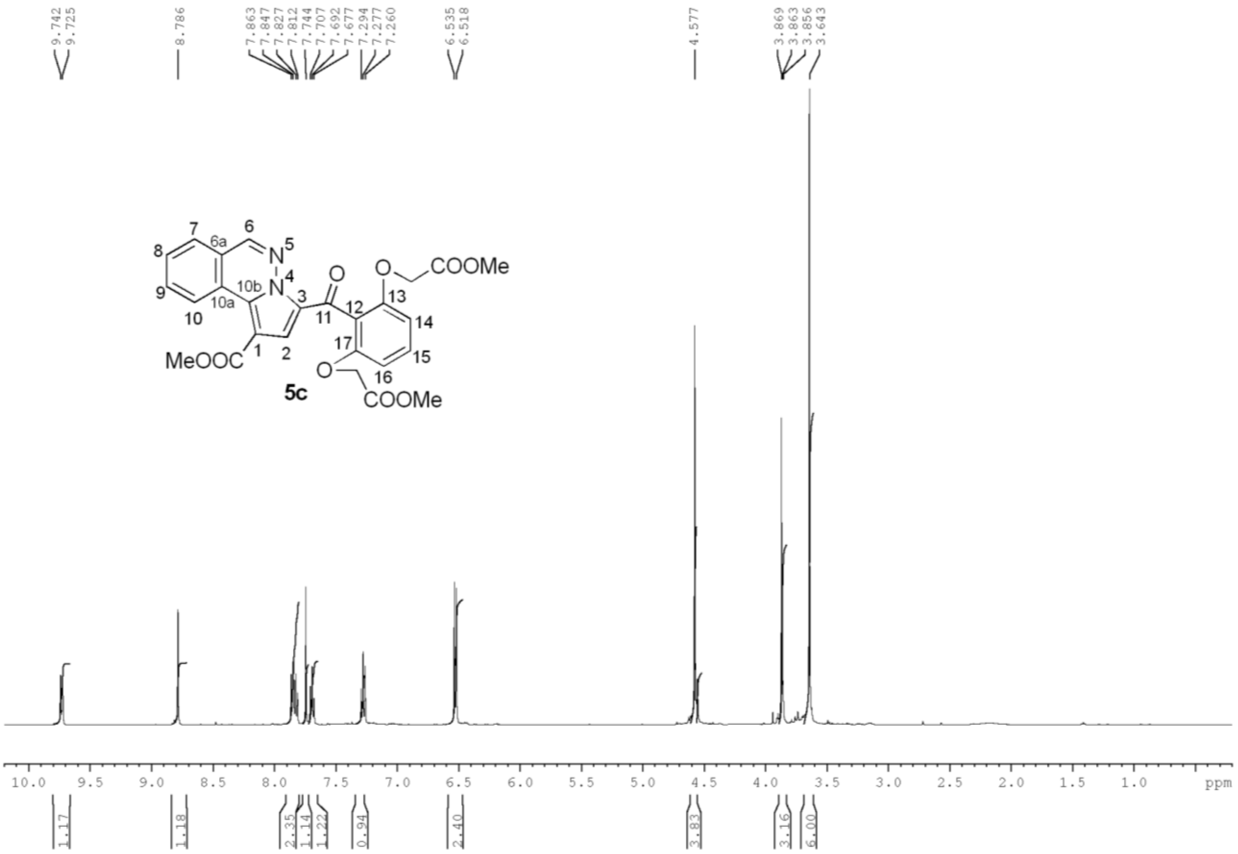

Figure S5: ^{1}H-NMR spectrum of the compound **5c.**

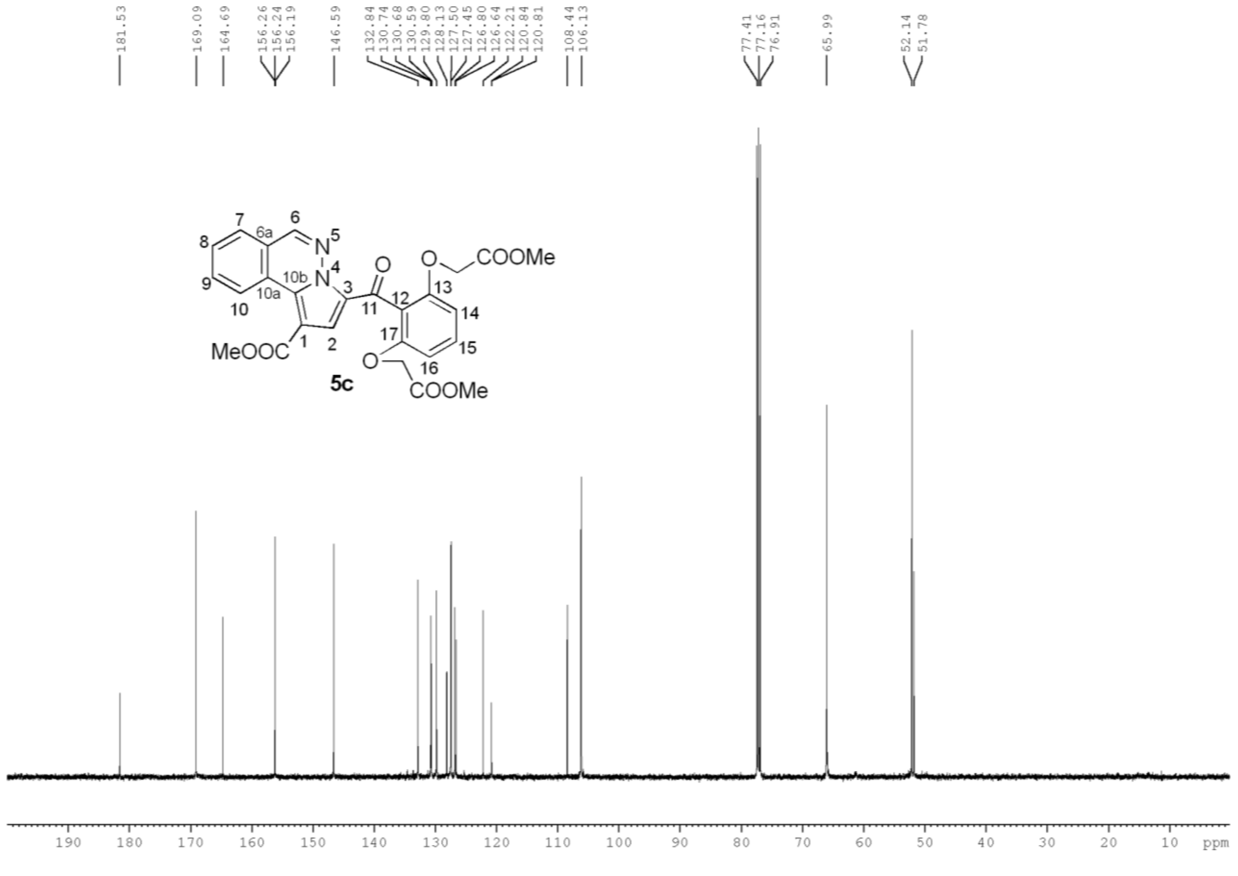

Figure S6: ^{13}C-NMR spectrum of the compound **5c.**

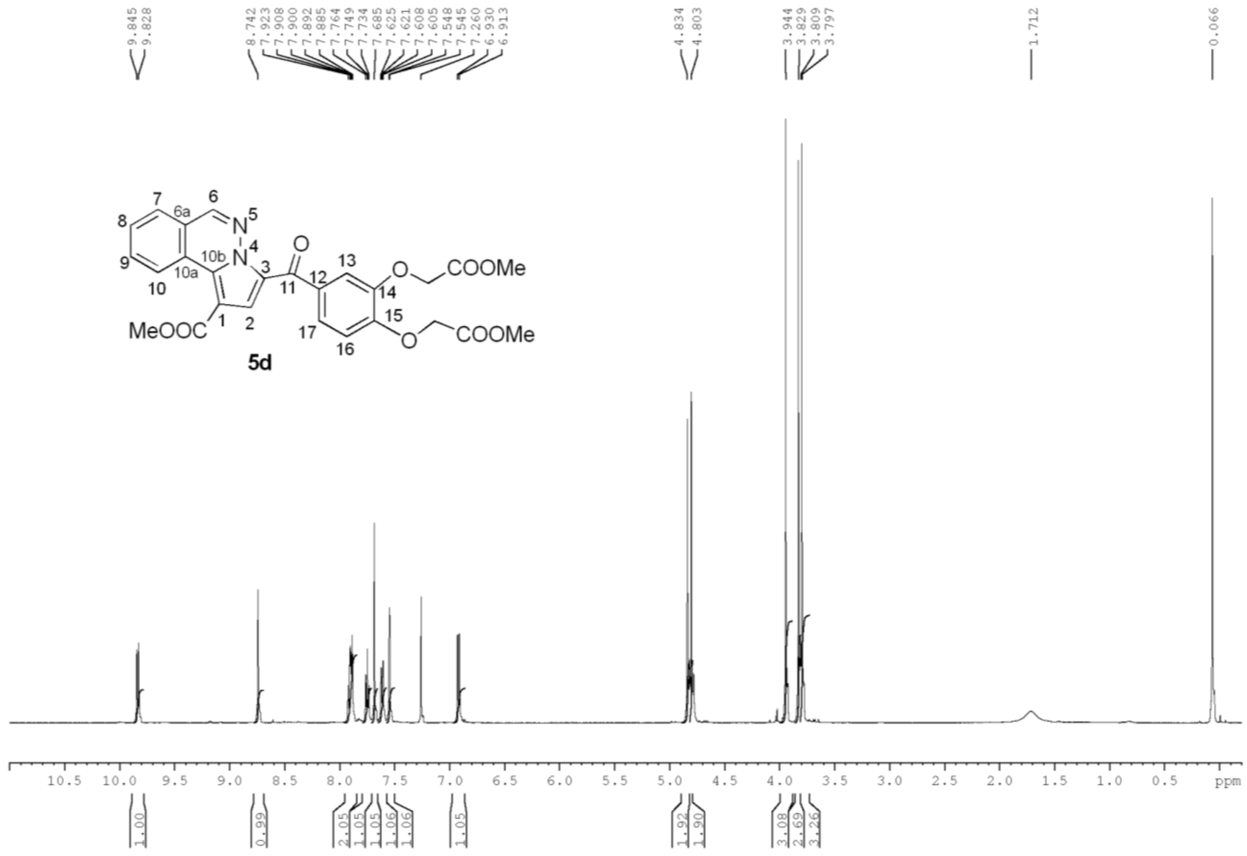

Figure S7: ^{1}H-NMR spectrum of the compound **5d.**

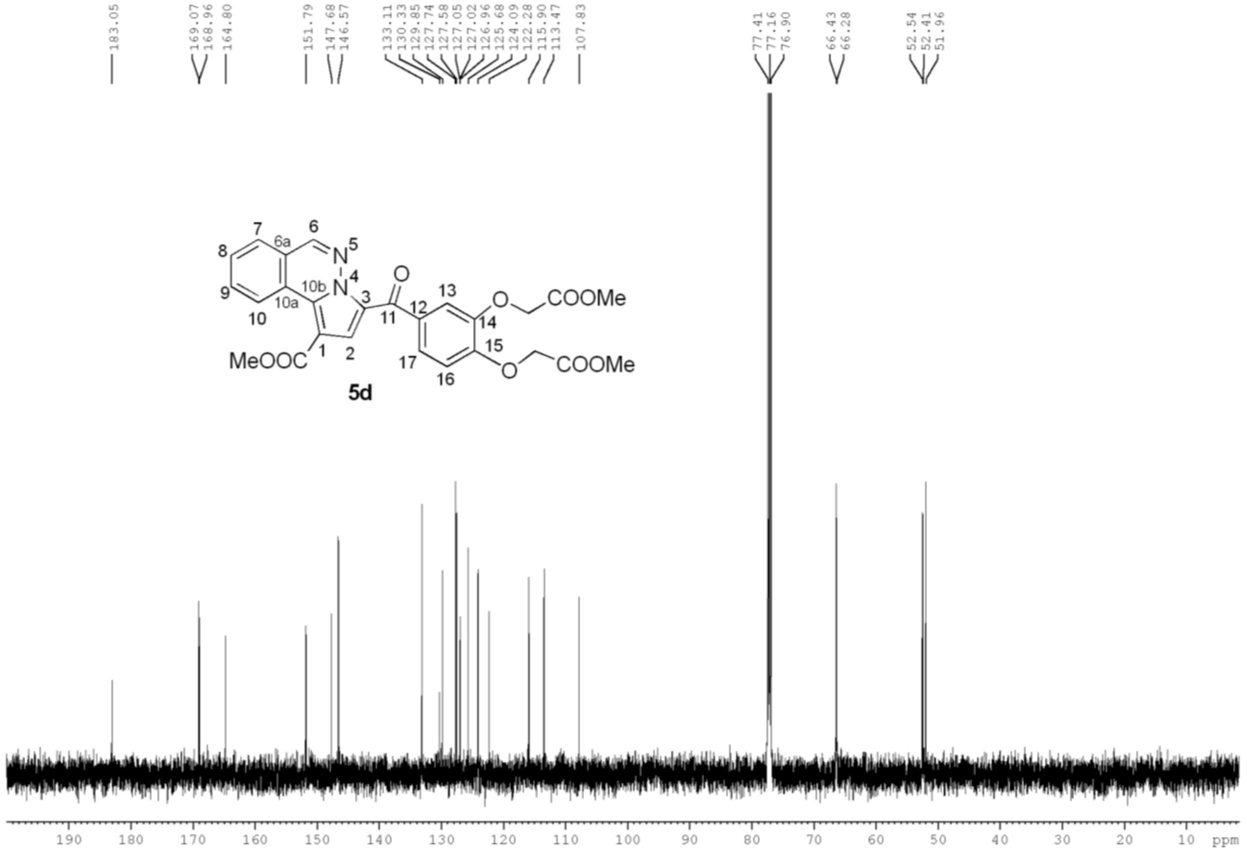

Figure S8: ^{13}C-NMR spectrum of the compound **5d.**

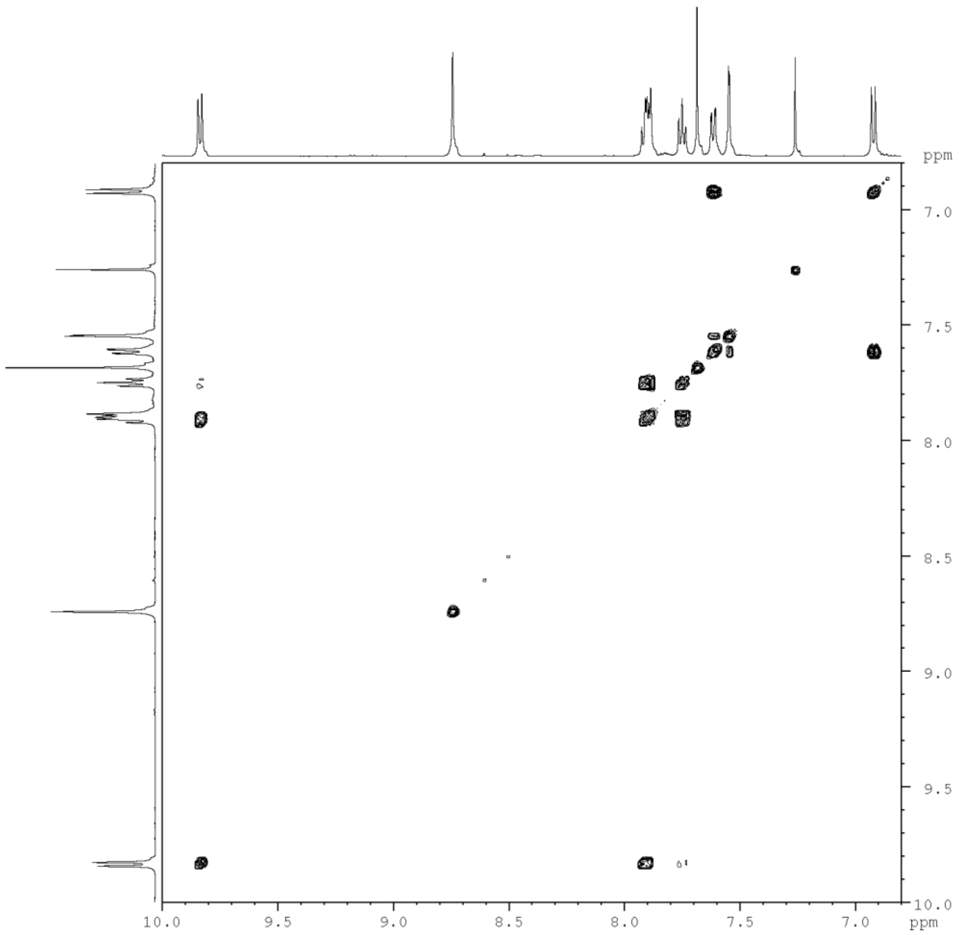

Figure S9: 2D-COSY 45 spectrum of the compound **5d.**

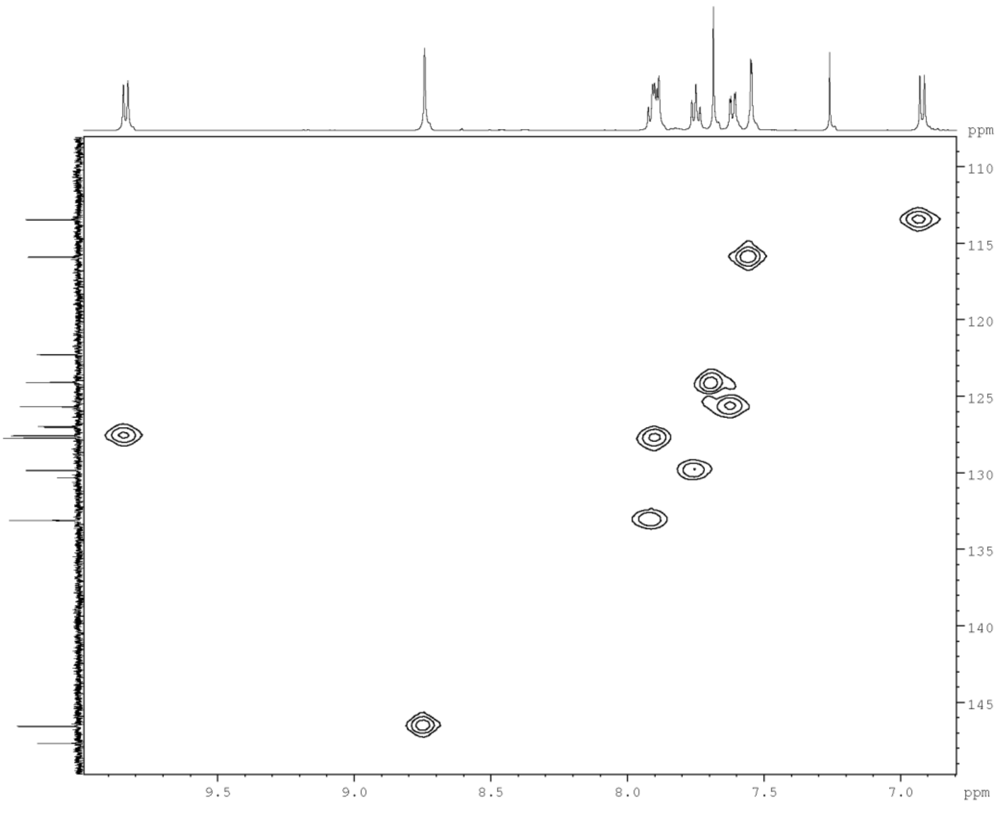

Figure S10: 2D-HETCOR (HMQC) spectrum of the compound **5d.**

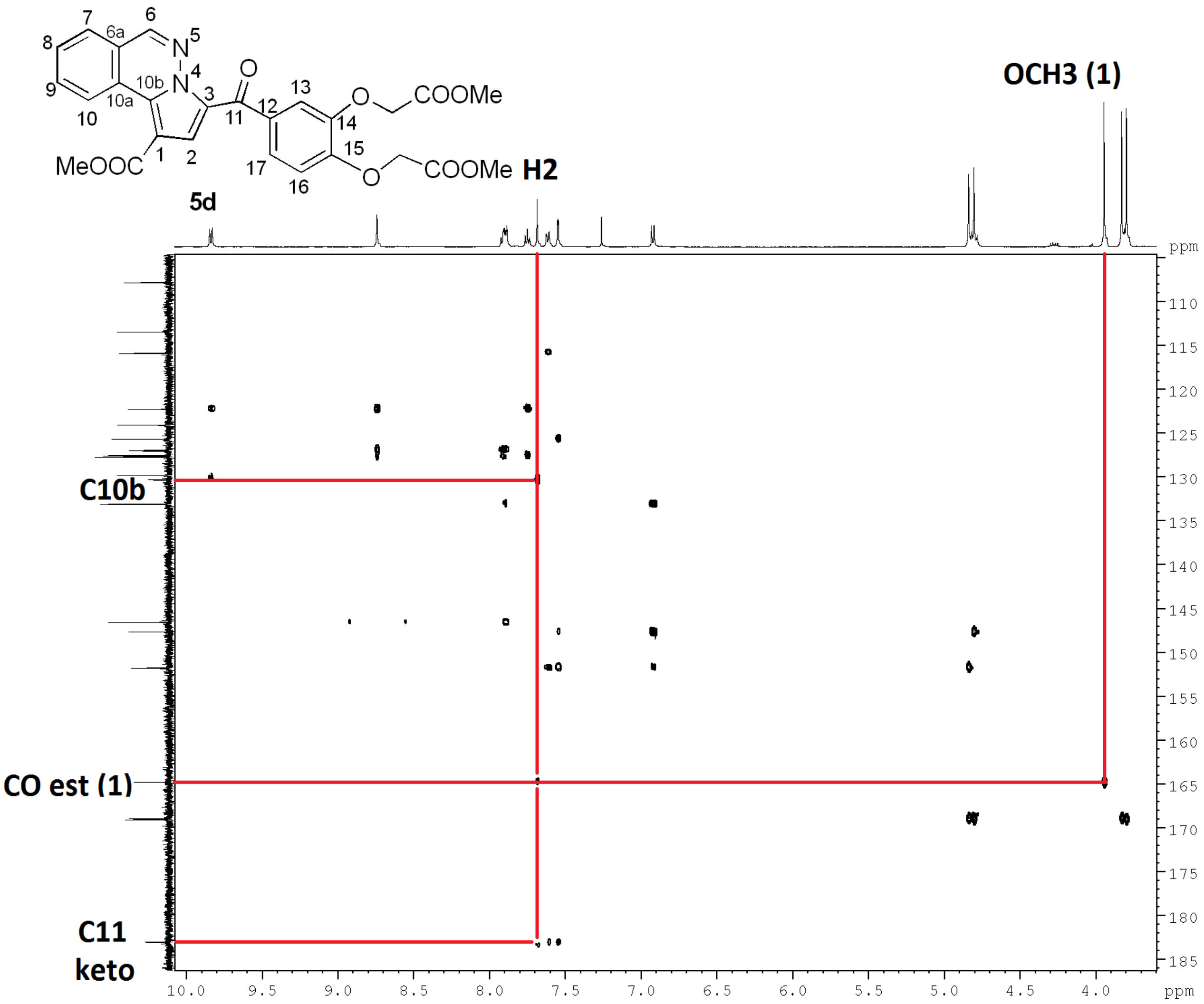

Figure S11: 2D-HETCOR (HMBC) spectrum of the compound **5d.**

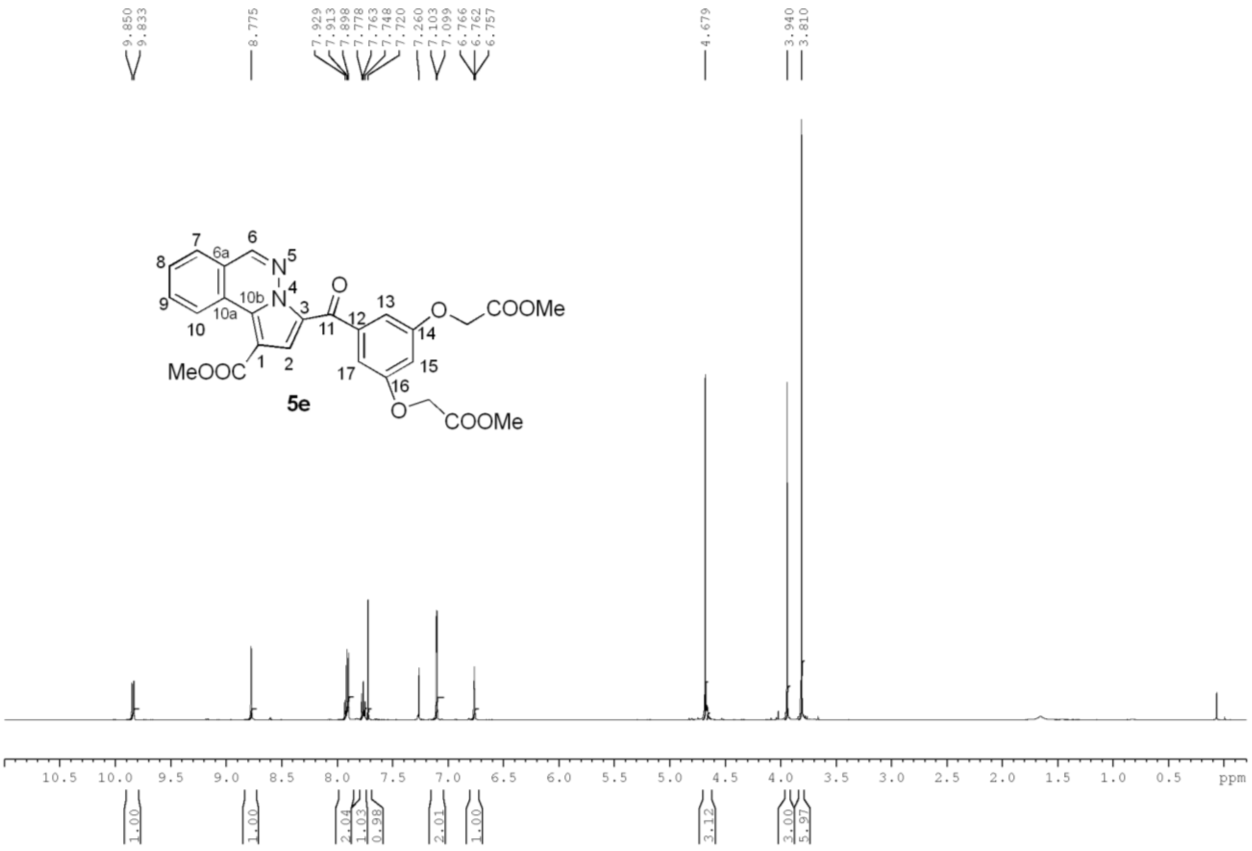

Figure S12: ^{1}H-NMR spectrum of the compound **5e.**

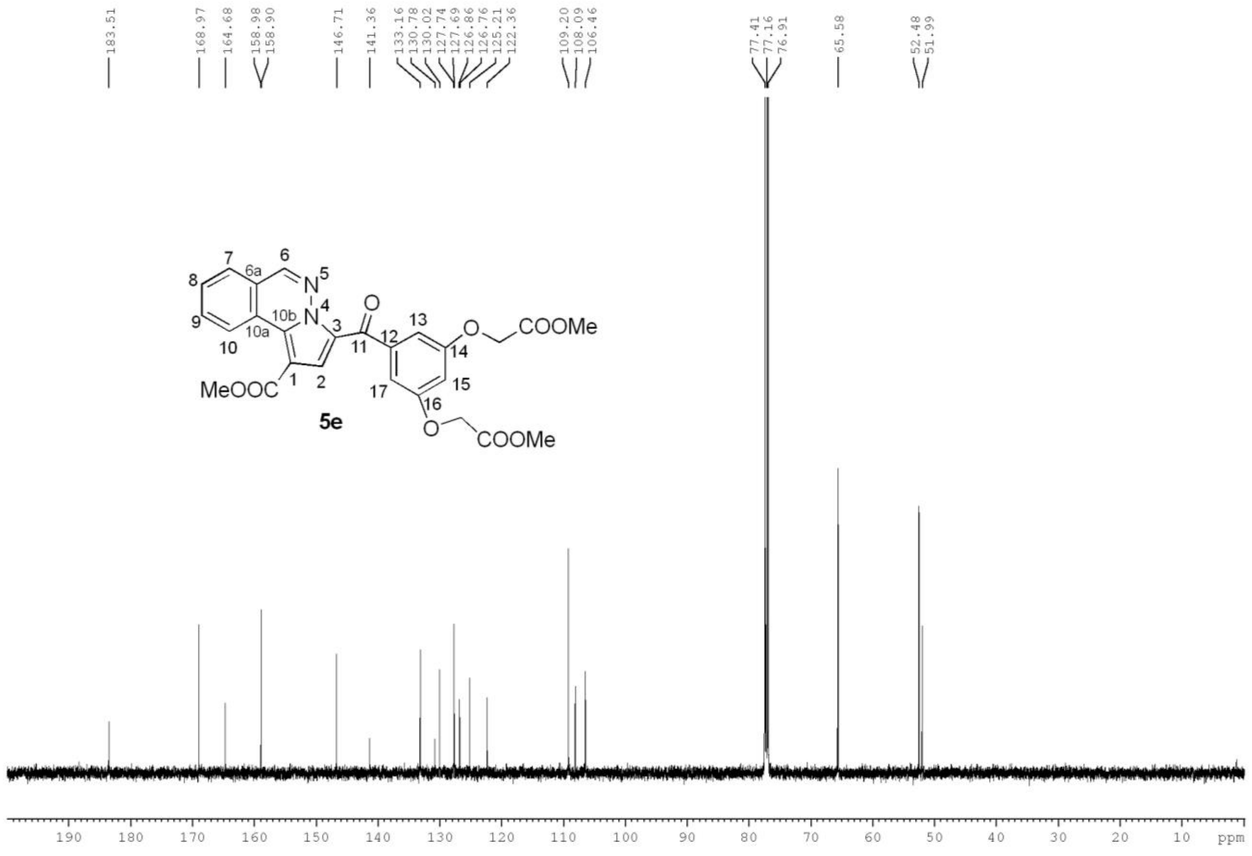

Figure S13: ^{13}C-NMR spectrum of the compound **5e.**

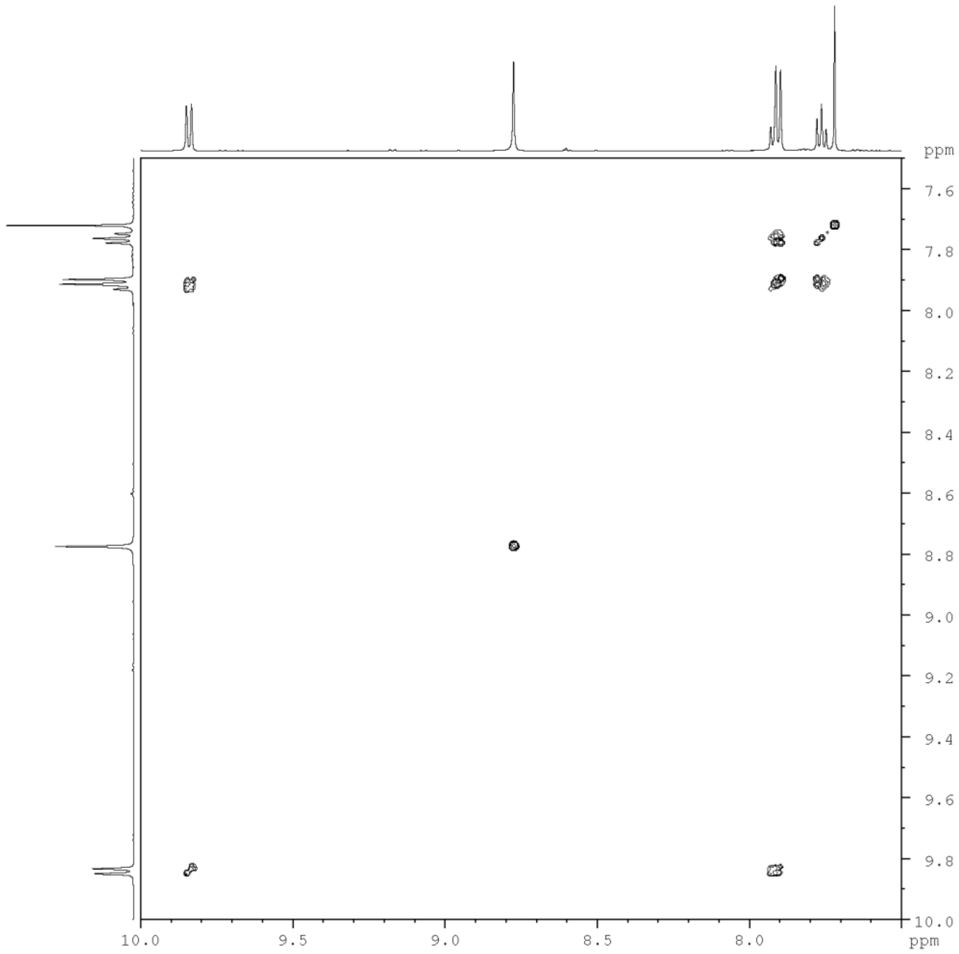

Figure S14: 2D-COSY 45 spectrum of the compound **5e.**

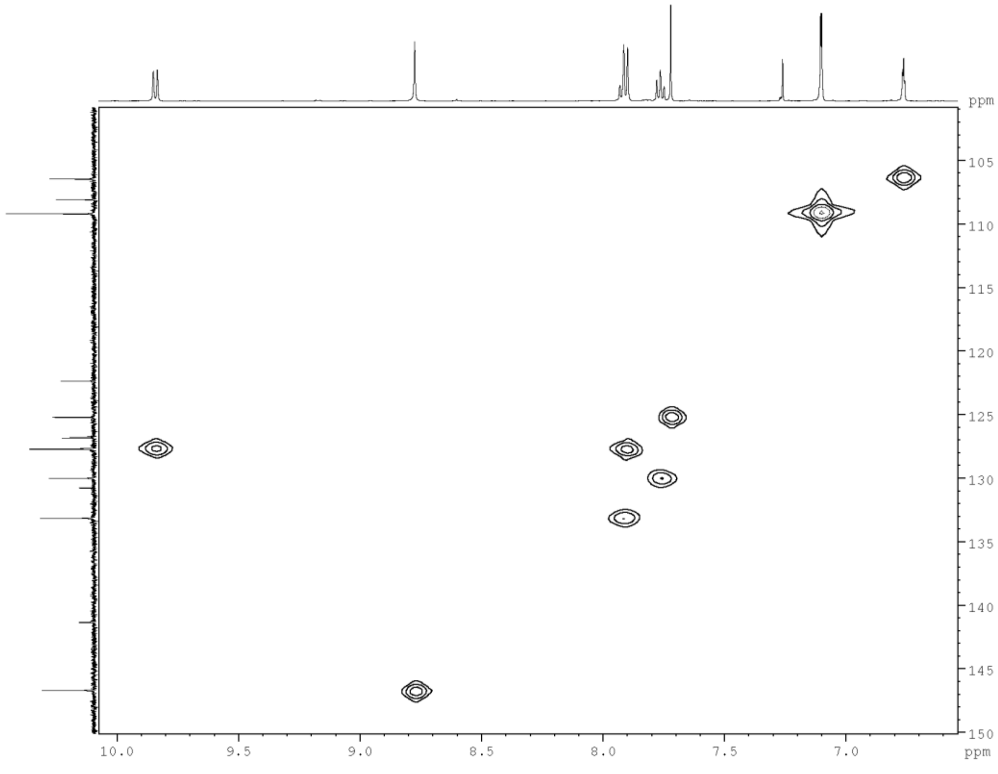

Figure S15: 2D-HETCOR (HMQC) spectrum of the compound **5e.**

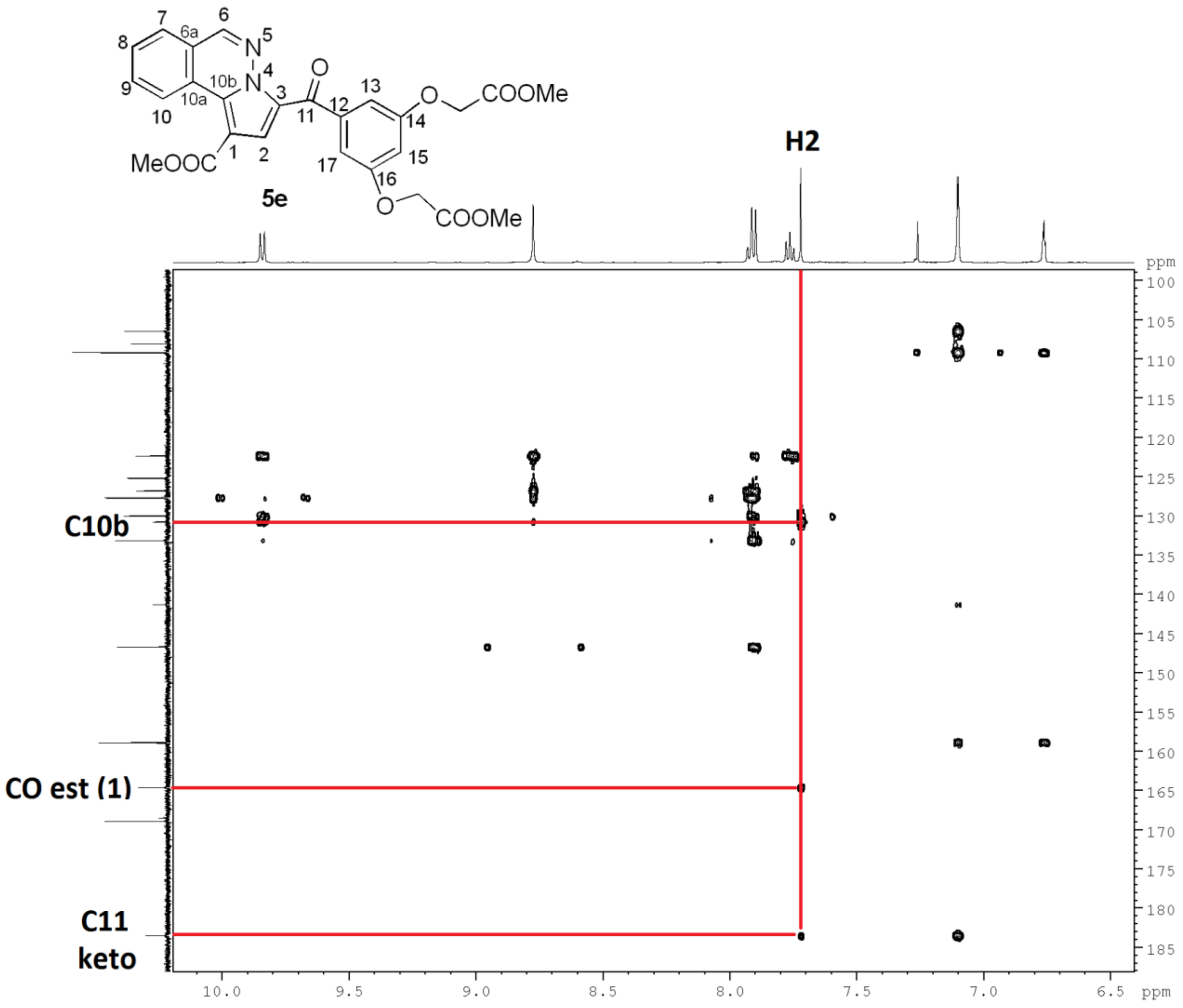

Figure S16: 2D-HETCOR (HMBC) spectrum of the compound **5e.**

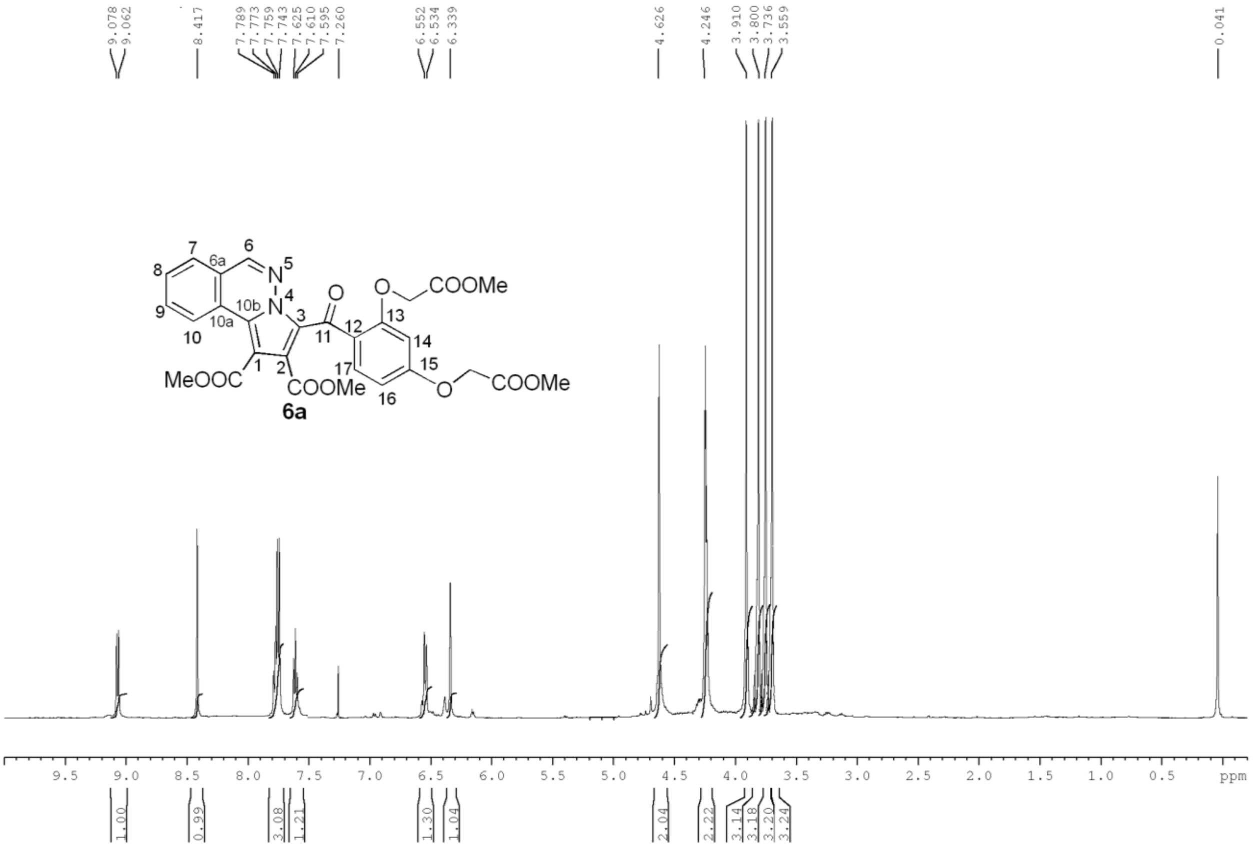

Figure S17: ^{1}H-NMR spectrum of the compound **6a.**

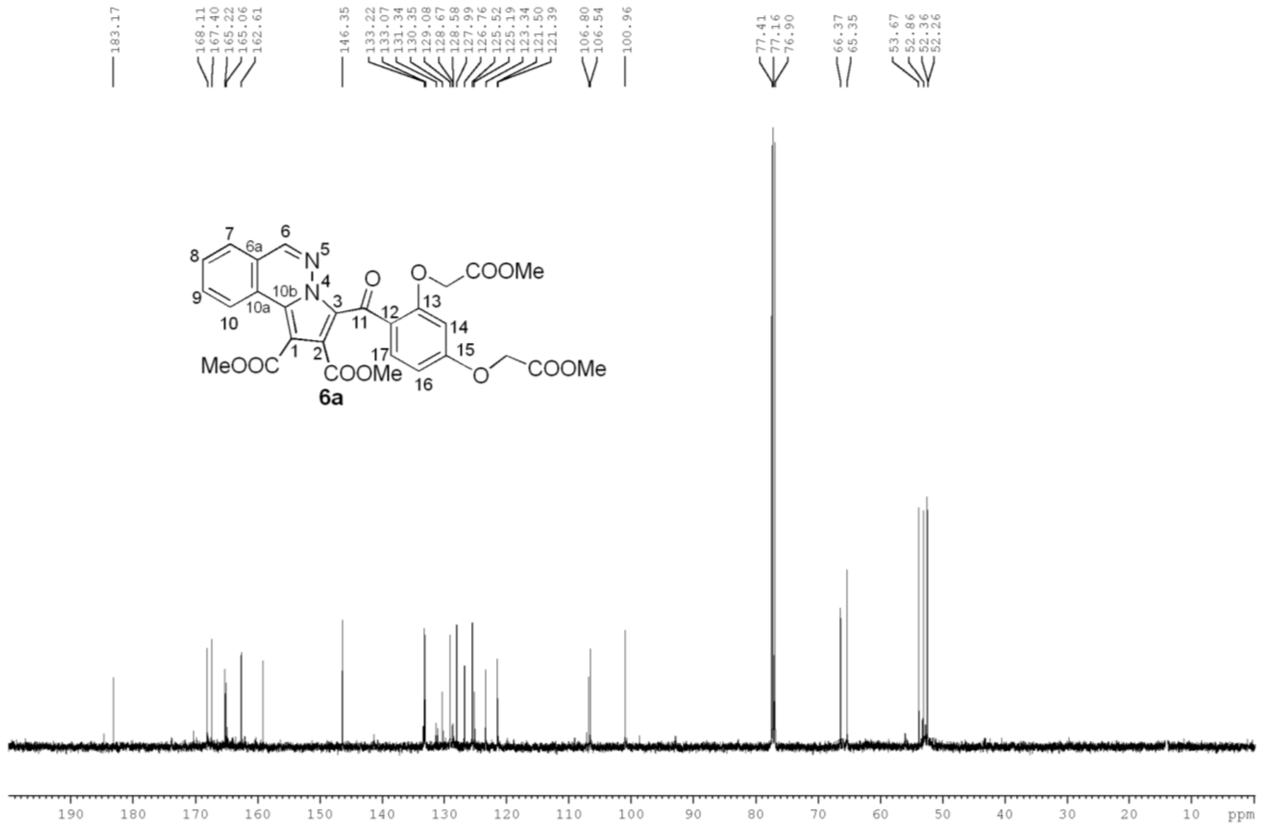

Figure S18: ^{13}C-NMR spectrum of the compound **6a.**

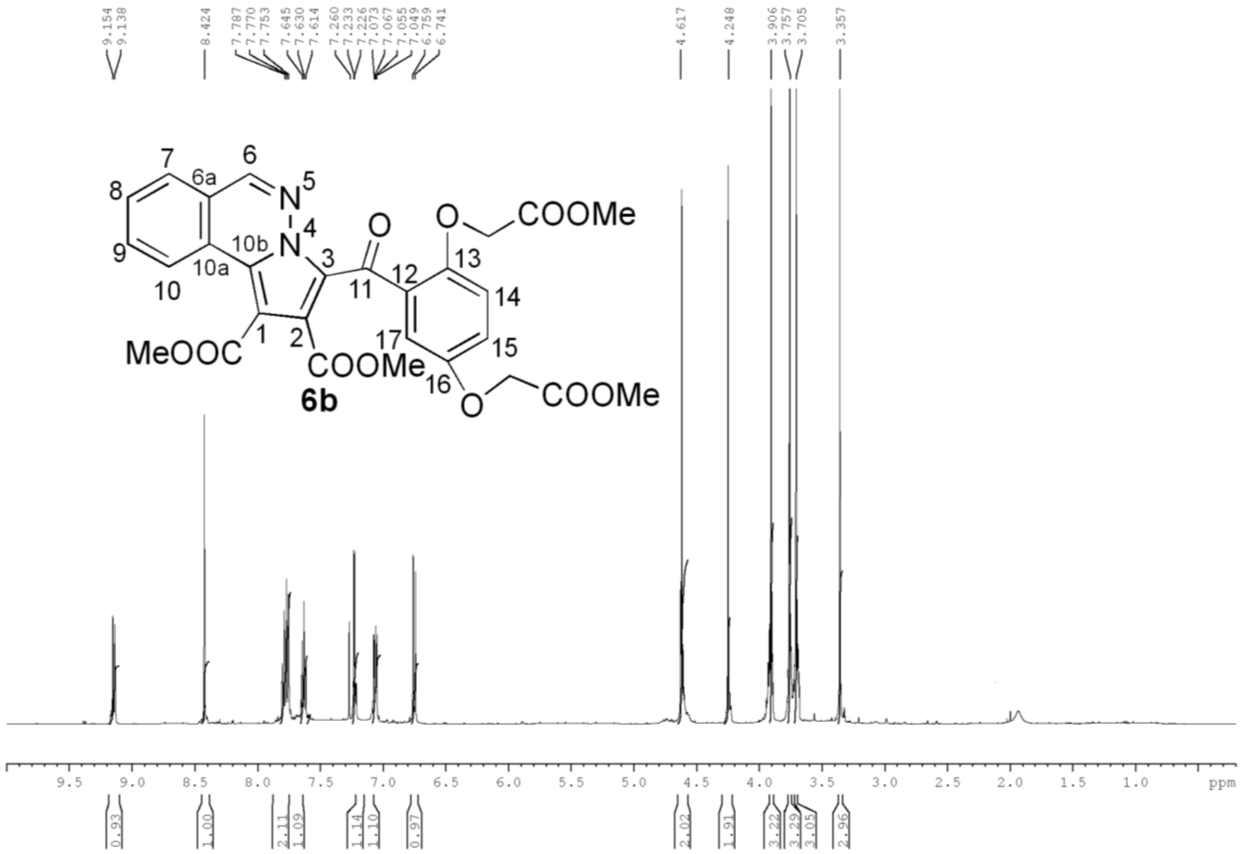

Figure S19: ^{1}H-NMR spectrum of the compound **6b.**

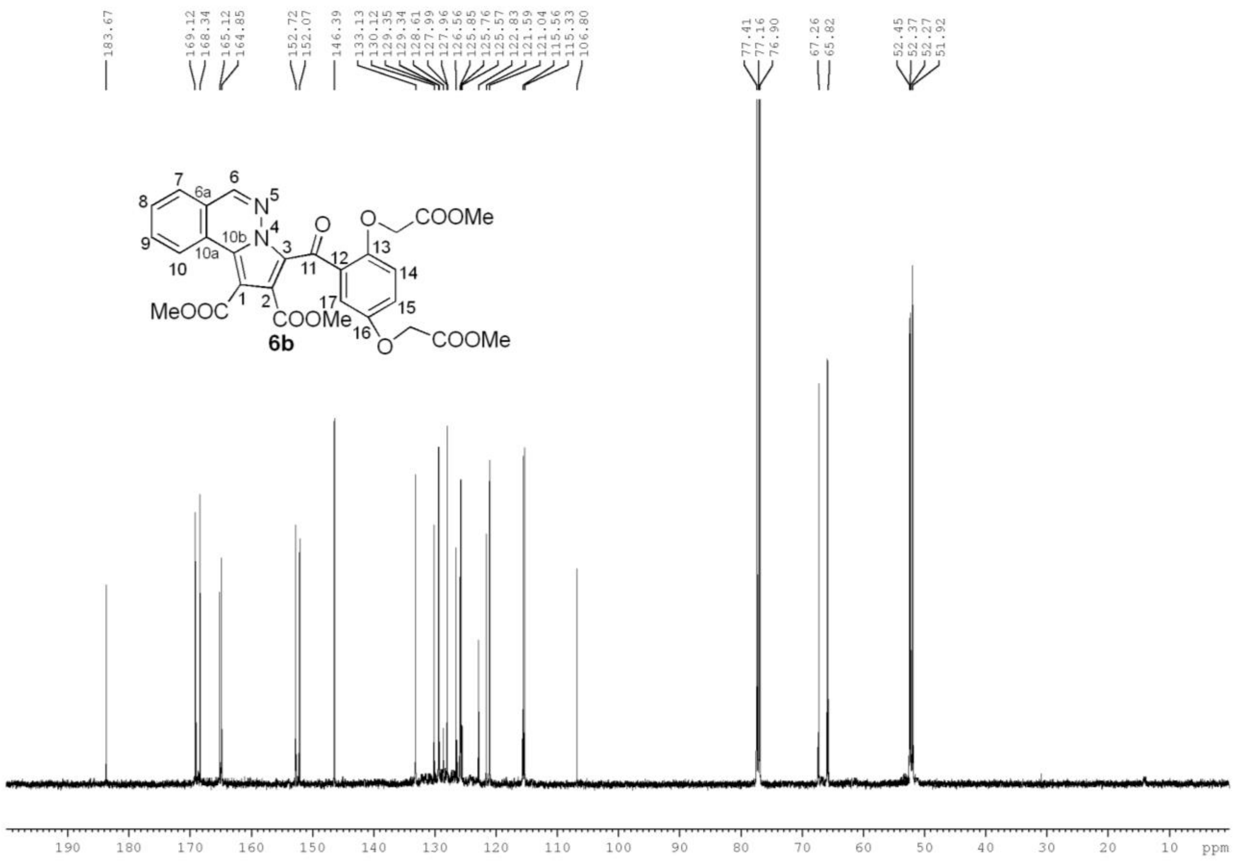

Figure S20: ^{13}C-NMR spectrum of the compound **6b.**

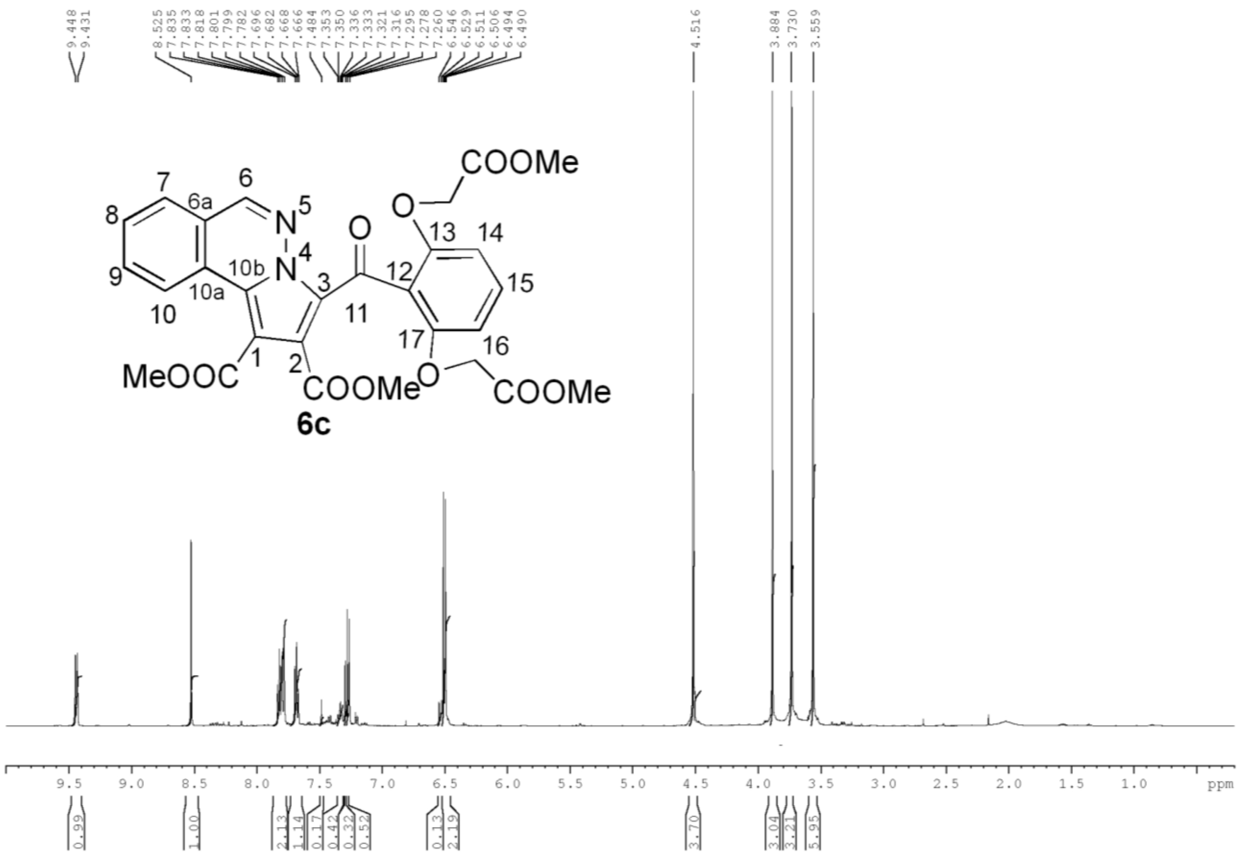

Figure S21: ^{1}H-NMR spectrum of the compound **6c.**

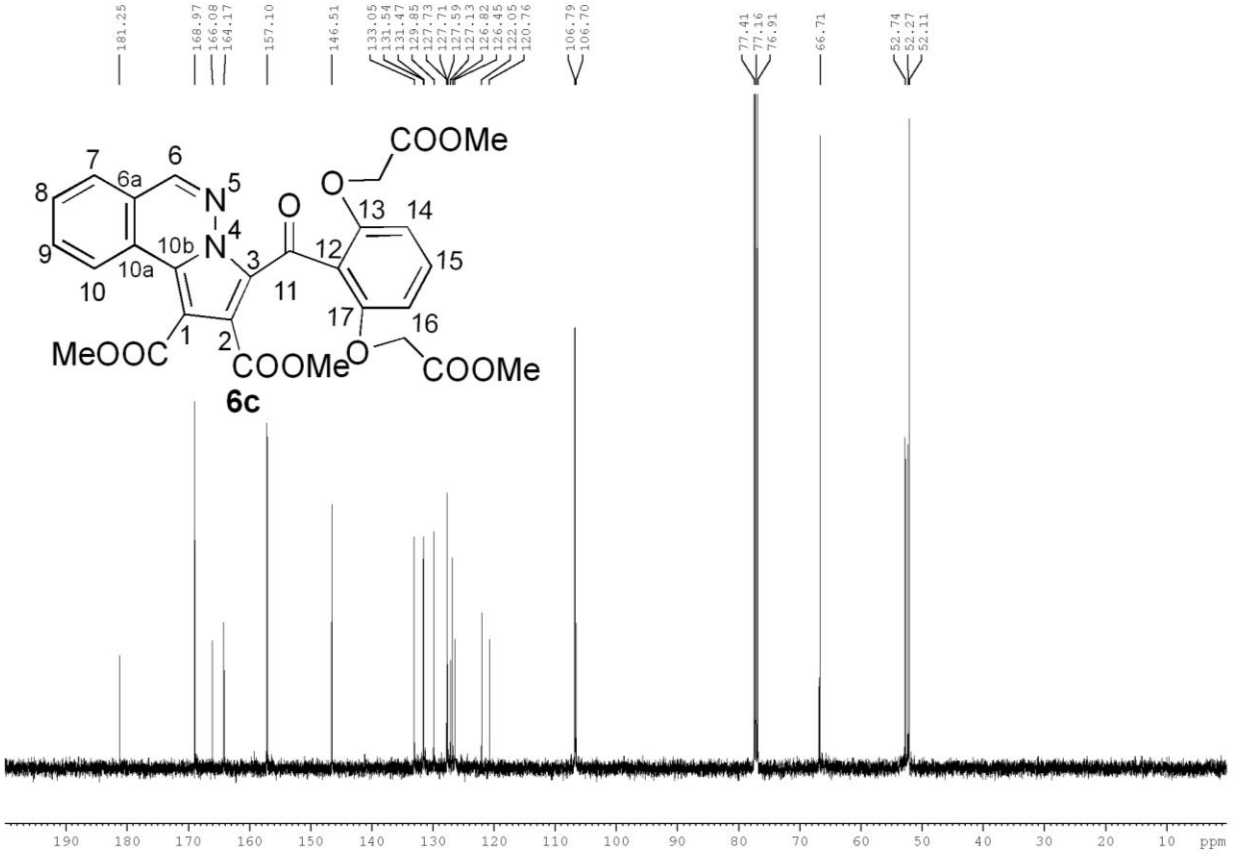

Figure S22: ^{13}C-NMR spectrum of the compound **6c.**

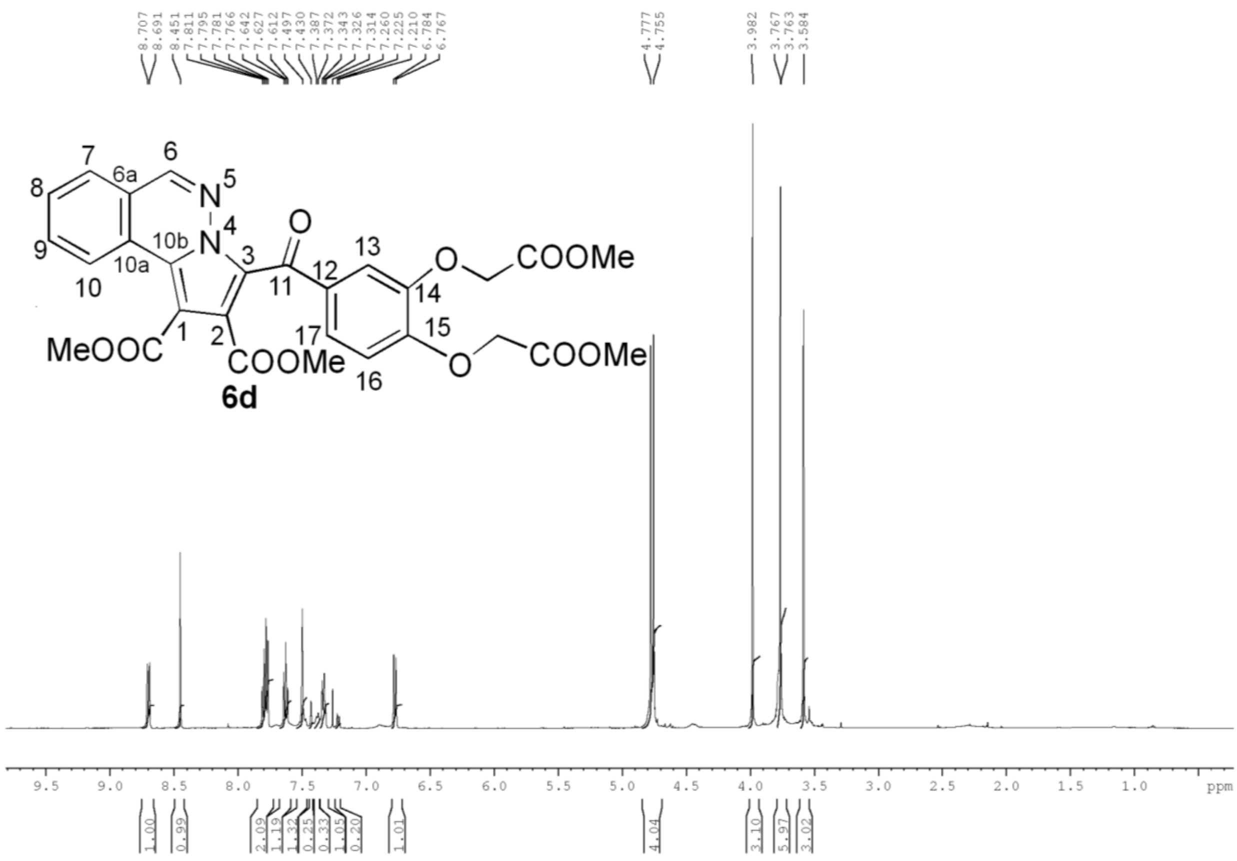

Figure S23: ^{1}H-NMR spectrum of the compound **6d.**

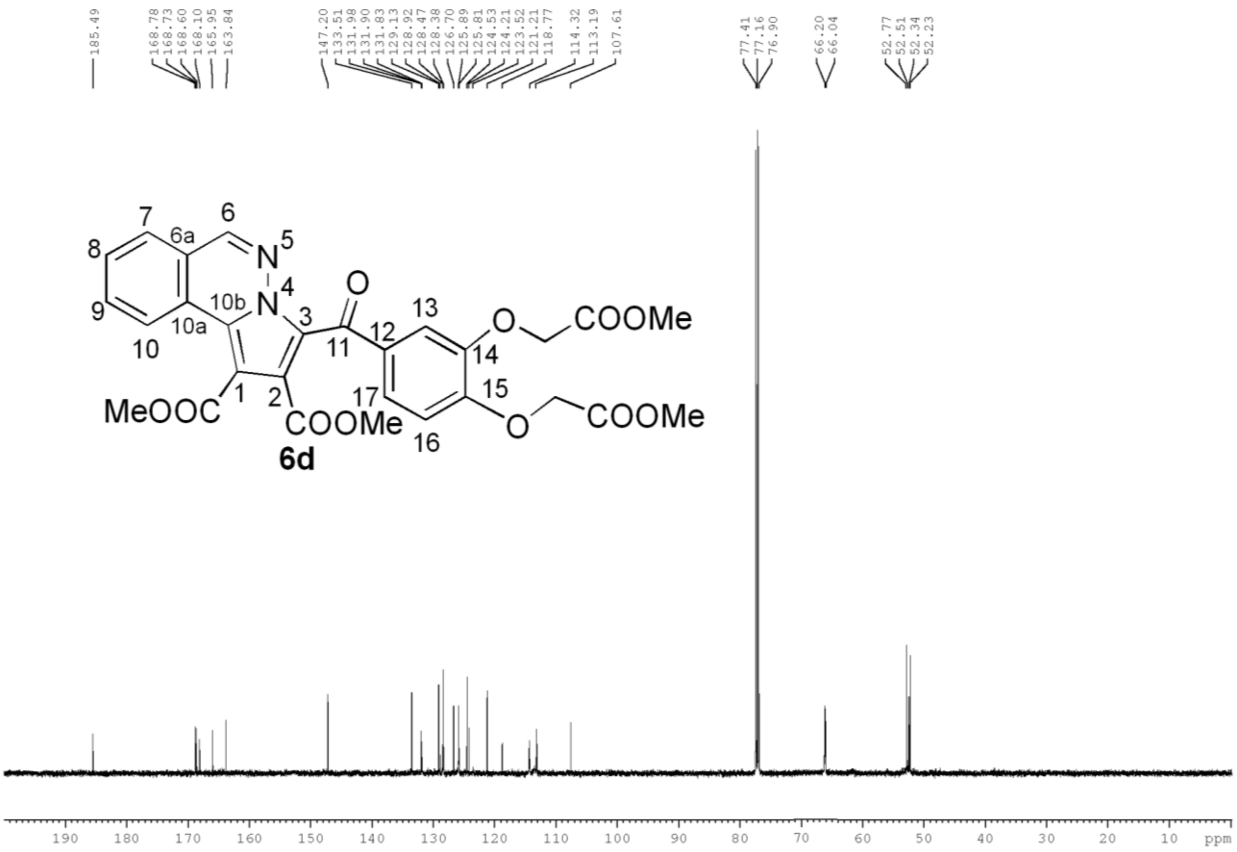

Figure S24: ^{13}C-NMR spectrum of the compound **6d.**

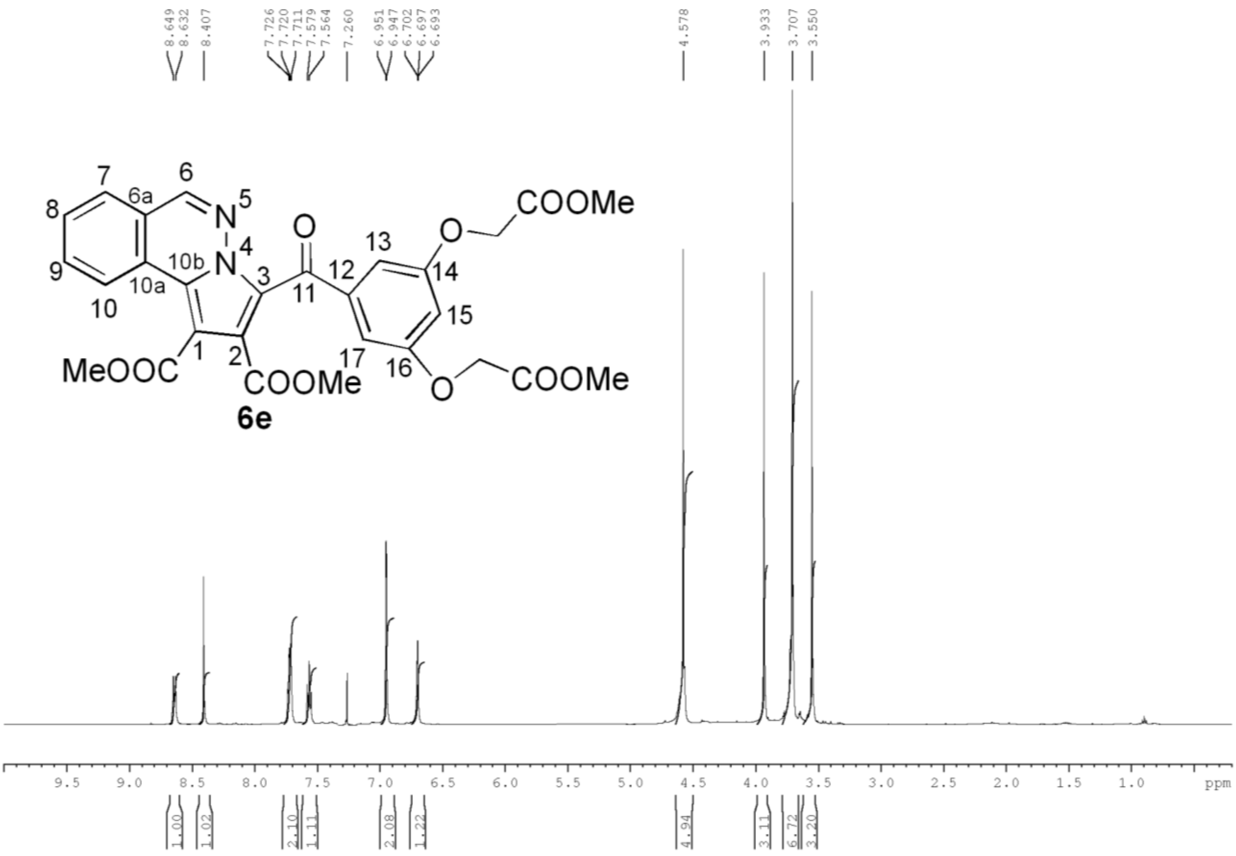

Figure S25: ^{1}H-NMR spectrum of the compound **6e.**

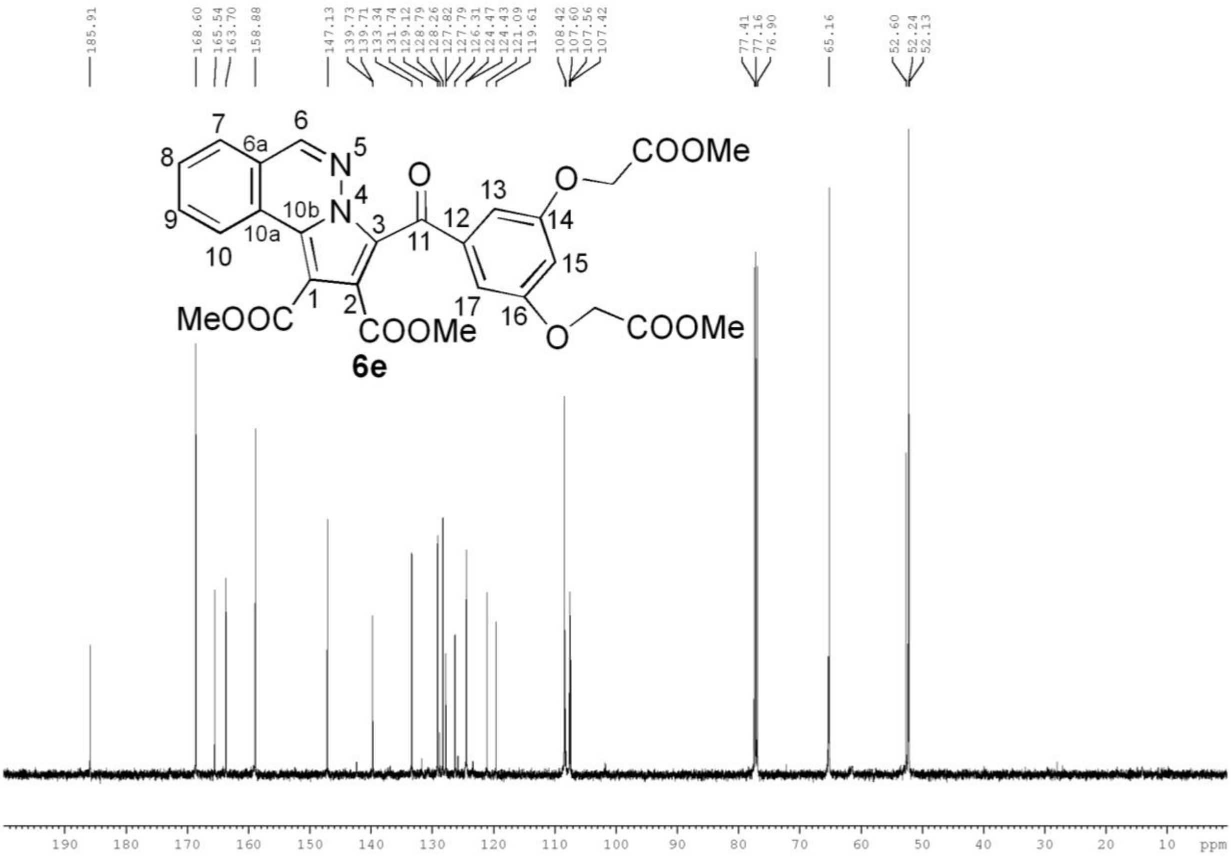

Figure S26: ^{13}C-NMR spectrum of the compound **6e.**
